# An Update on Obstructive Sleep Apnea for Atherosclerosis: Mechanism, Diagnosis, and Treatment

**DOI:** 10.3389/fcvm.2021.647071

**Published:** 2021-04-08

**Authors:** Jin Chen, Shu Lin, Yiming Zeng

**Affiliations:** ^1^Clinical Center for Molecular Diagnosis and Therapy, The Second Affiliated Hospital of Fujian Medical University, Quanzhou, China; ^2^Centre of Neurological and Metabolic Research, The Second Affiliated Hospital of Fujian Medical University, Quanzhou, China; ^3^Department of Cardiology, Southwest Hospital, Third Military Medical University, Chongqing, China; ^4^Department of Respiratory Medicine, The Second Affiliated Hospital of Fujian Medical University, Quanzhou, China

**Keywords:** obstructive sleep apnea, atherosclerosis, hypoxia, mechanism, treatment

## Abstract

The occurrence and development of atherosclerosis could be influenced by intermittent hypoxia. Obstructive sleep apnea (OSA), characterized by intermittent hypoxia, is world-wide prevalence with increasing morbidity and mortality rates. Researches remain focused on the study of its mechanism and improvement of diagnosis and treatment. However, the underlying mechanism is complex, and the best practice for OSA diagnosis and treatment considering atherosclerosis and related cardiovascular diseases is still debatable. In this review, we provided an update on research in OSA in the last 5 years with regard to atherosclerosis. The processes of inflammation, oxidative stress, autonomic nervous system activation, vascular dysfunction, platelet activation, metabolite dysfunction, small molecule RNA regulation, and the cardioprotective occurrence was discussed. Additionally, improved diagnosis such as, the utilized of portable device, and treatment especially with inconsistent results in continuous positive airway pressure and mandibular advancement devices were illustrated in detail. Therefore, further fundamental and clinical research should be carried out for a better understanding the deep interaction between OSA and atherosclerosis, as well as the suggestion of newer diagnostic and treatment options.

## Introduction

Obstructive sleep apnea (OSA) is known to commonly occur worldwide, with a recent dramatic increase in prevalence. Globally, an estimated 936 million and 425 million adults aged between 30 and 69 years experience mild-to-severe and moderate-to-severe OSA respectively (1). OSA is characterized by recurrent pauses in breathing during the sleep and results in upper airway collapse and intermittent hypoxemia (IH) (2). OSA is a systemic disorder and recognized as an independent factor for cardiovascular disease (3).

Atherosclerosis (AS) is a chronic disease of medium- and large-sized arteries leading to ischemic heart disease and cardiovascular disease (4). Over the past few years, many studies have shown a link between OSA and atherosclerosis, and sex-based differences were surprisingly observed. Female patients with OSA but not males were significantly associated with incident heart failure or death (5). Further, in a larger scale, female patients were more likely to have cardiovascular diseases compared with males (6). OSA causes endothelial dysfunction (7), unstable plaque characteristics (8) and further atherosclerosis (9). Similarly, IH also causes endothelial dysfunction (10) and accelerated atherosclerosis (11, 12). However, some reports argue that OSA may play a protective role in ischemic insult (13) and coronary occlusion (14) recently. Thus, the relationship between OSA and AS and related diseases needs to be further explored.

This review includes the clinical and fundamental research undertaken in the last 5 years describing the mechanism of OSA and its role in AS, as well as the latest research advances in the diagnosis and treatment of OSA and its influence on AS-related diseases.

## Mechanism

**Figure 1:**
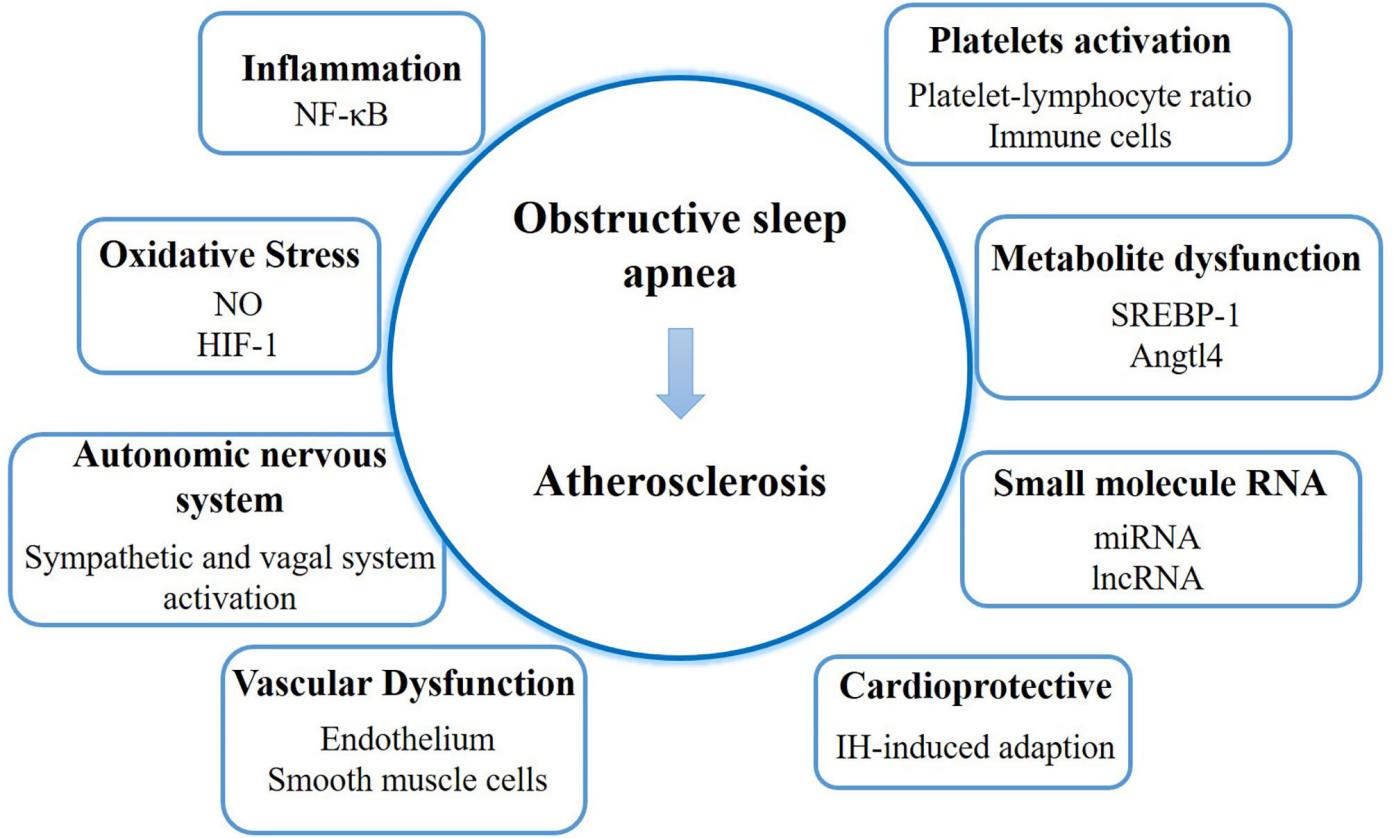
The mechanism and latest discoveries of the link between obstructive sleep apnea and atherosclerosis. NO, nitric oxide; HIF-1, hypoxia inducible factor-1; IH, intermittent hypoxemia.

The process of AS development in patients with OSA is complex. We discuss the underlying mechanism focusing on inflammation, oxidative stress, autonomic nervous system, vascular dysfunction, platelet activation, metabolite dysfunction, small molecule RNA, and the cardioprotective function (Figure 1).

### Inflammation

Since AS is a chronic inflammatory process, IH can produce a variety of inflammatory cytokines; therefore IH-mediated AS may be primarily due to the activation of inflammatory pathways (15). NF-κB is a key inflammation pathway that has been extensively studied.

Some reports illustrated the development of AS when NF-κB pathway was blocked. Mice with apolipoprotein E deficiency and knocked down NF-κB p50 showed higher serum levels of TNF-α, IL-6, and cholesterol and more pronounced atherosclerotic lesions than mice with apolipoprotein E deficiency alone (16). When IKKβ, a regulator of NF-κB, was deleted in mice, IH-mediated pulmonary artery atherosclerosis was abolished (17). Another study utilized the overexpression of IkBα, an inhibitor of NF-κB, only in mice endothelial cells and showed a decline in developing atherosclerotic lesions. IkBα overexpression resulted in the suppression of E-selectin and vascular cell adhesion molecule-1 (VCAM-1) and inhibited the NF-κB pathway. Hence, AS was weakened under IH exposure (18). Collectively, these studies supported IH-induced AS via the NF-κB pathway.

Upstream of NF-κB has also been discussed in the IH-mediated AS. Besides common molecules such as IL6, TNF-α, and p38 MAPK, TLR4/ NF-κB was discovered as a new therapeutic target. Increased TLR4 expression was observed both in IH-treated mice (19) and OSA patients (20). When TLR4 was inhibited in mice, enhanced NF-κB pathway, and augmented atherosclerotic plaque loads were diminished (19). When TLR4 was depleted in mice, smaller intima-media aorta thickness was observed than in normal mice (21). Thus, TLR4/NF-κB plays an important role in inflammation, indicating alternative therapeutic option.

### Oxidative Stress

Oxidative stress is another fundamental mechanism contributing to the cardiovascular diseases in OSA. It has been shown that IH triggers the activation of NADPH oxidase and increases leukocytes oxidation, resulting in lipid peroxidation and isoprostane formation. Meanwhile, the function of nitric oxide in endothelial cells is inhibited because of reduced endothelial nitric oxide (NO) synthase and regulation of asymmetric dimethylarginine (22). All these processes are involved in the formation of AS and related cardiovascular diseases. Further, other proteins were found to participate in AS formation in IH, such as, non-muscle myosin light chain kinase which was discovered in the mouse model (23). Interestingly, recent studies have also shown that IH and oxidative stress can activate the protective mechanism of endothelial cell-colony forming units in a cell model extracted from healthy volunteers, which is closely linked with vascular function and maintains vascular health (24).

Hypoxia inducible factor-1 (HIF-1) is a transcription factor that promotes genes involved in adaptation to insufficient oxygen and hypoxia environment. ROS induced by desaturation-reoxygenation under IH could up-regulate HIF-1 activity through complex processes (25). In brief, ROS activates phospholipase C, followed by calcium-calmodulin kinase and protein kinase C activation. Protein kinase C stimulates the mTOR-dependent production of HIF-1α and inhibited PHD-dependent degradation of HIF-1α (26). HIF-1 promotes numerous adaptive genes such as, endothelin-1, which could destroy the cardiovascular system (25). It is reported that the profiling of gene expression including HIF-1 in skin biopsies from OSA patients could possibly be utilized to predict cardiovascular risk (27).

### Autonomic Nervous System

The autonomic nervous system including the sympathetic and vagal system is altered in patients with OSA. In brief, IH stimulates the peripheral chemoreceptor and activates sympathetic nervous system, followed by an increase in renin, angiotensin II, and aldosterone, and the enhancement of vasoconstrictor activity. At the same time, impaired baroreflex and reduction of NO resulted in the increase of endothelin and receptor as well as intracellular calcium sensitivity (28). In these ways, IH induces abnormal vasoconstriction and elevates systemic blood pressure.

### Vascular Dysfunction

Disruption of vascular endothelium homeostasis in OSA is triggered by inflammation and oxidative stress of endothelial cells (29) and is modestly linked with subclinical atherosclerotic coronary artery disease (CAD) (30). Also, individual patient data meta-analysis showed that severe OSA is independently linked with an increased endothelial dysfunction (31). Non-muscular myosin light chain kinase was discovered to have been involved in IH-induced endothelial dysfunction through the secretion of IL-6, NO production, and acetylcholine in human aortic endothelial cells (32). Besides, the endothelial barrier function was also destroyed by phosphorylated ERK and JNK in human lung microvascular endothelial cells (33). Some protective measures have recently been investigated to improve endothelial dysfunction. Infliximab and glutathione, served as anti-inflammatory and antioxidant treatment, were shown to inhibit the vascular injury process from mouse models (34). *Rhodiola Crenulata* extract might protect the damage of human umbilical vein endothelial cells through the AMPK and ERK pathway (35).

IH triggers excessive proliferation of vascular smooth muscle cells, which play important roles in AS progression (36). It is reported that the production of IL-6 induced the upregulation of epiregulin, which contributed to the proliferation of smooth muscle cells (37).

### Platelet Activation

Patients with OSA suffer persistent platelet activation, as a consequence of increased sympathetic activity, inflammation, and endothelial dysfunction, which leads to AS lesions (38). Platelet activation leads to alteration in their shape and the phospholipid bilayer, resulting in the stimulation of coagulation factors and upregulation of surface receptors and adhesion molecules, enabling them to interact with other cells. The platelet-lymphocyte ratio is observed to be associated with OSA severity regardless of OSA progression, indicating that it may serve as an independent marker (39). In addition, platelets were reported to serve as immune cells participating in the pathophysiology of autoimmune disorders (40). Persistent platelet activation induces constant production of pro-inflammatory and proatherogenic substances, infiltration of immune cells in the endothelium and further the progression of AS plaques.

### Metabolite Dysfunction

Patients with OSA have abnormal glucose and lipid metabolism, contributing to the generation of AS (41, 42). The functional proteins involved in glycolipid metabolism in IH-mediated AS has garnered considerable research interest. A recent study described SREBP-1 signaling in the aorta, skeletal muscle, and liver, considering the synergistic effect of both IH and abnormal glucose metabolism. It was found that SREBP-1c and FAS increased, while IRS-1 and its phosphorylation decreased, thereby promoting AS *in vitro* and *in vivo* (43). In addition, augmented angiopoietin-like 4 in OSA via HIF-1 also played an important role in abnormal IH-induced lipid metabolism contributed to AS formation (11, 44).

### Small Molecule RNA

Small molecules RNAs such as miRNA, mRNA, and lncRNA provide detailed information about AS development in patients with OSA miRNAs from plasma exosomes were identified *via* arrays, and differentially expressed miRNAs would describe the altered endothelial function accounting for the mechanism of cardiovascular morbidities in OSA (45). Another report depicted the profiling of mRNAs and lncRNAs in the aorta when treated with IH. The description of this system may provide potential candidates for future research on IH-induced AS (46). In addition, its unique RNA was also explored to understand its function. It was reported that miR-193a-3p impaired human umbilical vein endothelial cells under IH exposure *via* Fas apoptotic inhibitory molecule 2 (47). Similarly, miR-146a-5p aggravated IH-induced heart myoblast injury through an X-linked inhibitor of apoptosis protein (48). However, both studies were only conducted *in vitro* only. Taken together, RNA showed its potentiality in inspecting the mechanism and treatment therapy of cardiovascular diseases in OSA.

### Cardioprotective Pathway

More recently, increasing evidence has shown that intermittent hypoxia can trigger an adaptation in cardiovascular system (49), such as, the resistance to an ischemic insult (50) and attenuation of ischemic brain injury (51). In acute coronary syndrome, myocardial infarct size significantly decreased in patients with OSA compared to in patients without, as determined by measuring peak, and area of serial cardiac troponin I levels (52). These results were consistent with the study that patients with OSA and acute myocardial infarction showed lower peak troponin-T levels (53). However, these results should interpreted with cautions because of some limitations (13). First, the measurement of infarct size can hardly be accurate and would be affected by multiple factors. Second, the number of cases recruited was small, and the results need to be verified in a larger study. Third, the patient group had a large dynamic range of troponin levels with approximately a 100-fold difference. Thus, although the clinical protective effect of IH cannot yet be clearly established, these results indicated that OSA might upregulate the cardioprotective pathway and promote adaptive process.

## Update on Diagnosis

The apnea-hypopnea index (AHI) is widely used in the identification and classification of clinical OSA. The gold standard of AHI detection is measured by overnight polysomnography in the sleep laboratory, and values of 5–15, 15–30, and >30 are defined as mild, moderate, and severe OSA, respectively (54). Further, polysomnography could give mechanistic indices such as loop gain, arousal threshold, and pharyngeal collapsibility with validated methods, and it was able to distinguish sex differences in patients with OSA (55). However, cost and accessibility limit the application of polysomnography in all settings. Recently, other methods such as, home sleep apnea testing and questionnaires have been further investigated to diagnose patients with OSA and those with AS. Moreover, new algorithms have also emerged to ensure a much more convenient diagnosis (Table 1).

**Table 1:** Update on diagnosis.

**Method**	**Key points in OSA**	**References**	**Key points in patients with OSA and AS - related diseases**	**References**
Home sleep apnea testing	An alternative for diagnosis of moderate to severe OSA	(56, 57)	The strong correlation coefficients of HSAT and polysomnography in OSA with heart failure	(58, 59)
Questionnaires	STOP-Bang questionnaire showed the most sensitivity and diagnostic odds ratio and was used to estimate the severity of OSA	(61, 62)	STOP-Bang questionnaire showed the most sensitivity with low specificity in OSA with cardiovascular diseases	(63, 64)
New algorithm	Provided an alternative approach to screen suspected patients with OSA	(66–68, 70)	Algorithm constituted by optimized cardiovascular signals provided a good representation of AHI	(69)

Home sleep apnea testing (HSAT), also known as out-of-center sleep testing, portable sleeping monitoring, and portable monitoring, is an alternative for the diagnosis of moderate-to-severe OSA (56). However, according to the statement of the American Academy of Sleep Medicine, the decision should not be based solely on the automatically scored HSAT data (57). More recently, HSAT has been used to assess the relationship between sleep apnea and cardiovascular diseases. The results showed that a portable ApneaLink device could be used to identify patients with heart failure with sleep apnea (AHI≥15 and <5 events/h), which contained both obstructive and central apneas (58). In another study, researchers distinguished the central and obstructive AHI values in another study, and also found a strong correlation coefficient between HSAT and polysomnography, which indicated a possible diagnosis (59). However, HSAT does not appear to have reduced the cost, as it is only 10% lower than the cost of polysomnography for the provider (60).

To screen patients with OSA, researchers developed questionnaires such as the Berlin questionnaire (BQ), Epworth Sleepiness Scale (ESS), STOP questionnaire (STOP), and STOP-Bang questionnaire (SBQ). It was found that SBQ showed the most sensitivity and diagnostic odds ratio among these four questionnaires and was used to estimate OSA severity (61, 62). However, it remained obscure whether a sleep disorder with cardiovascular diseases could be diagnosed by questionnaires, as typical sleep-disordered symptoms may not be observed in these patients. According to the results of 89 patients with cardiovascular diseases, ESS showed no association with sleep disorder while BQ showed the sensitivity of 73% with a specificity of 42%. SBQ showed the most sensitivity (97%) with low specificity (13%) (63). Besides, insufficient specificity was observed in OSA with atrial fibrillation (64), and poor correlation was discussed in OSA with stroke (65). Taken together, to better reflect the phenotypes in patients with co-morbid conditions, the questionnaires need to be improved.

A new algorithm was developed for identifying OSA. Electrocardiograph-based algorithm, such as, support vector machines (66), Kernel density classifier (67), and convolutional neural network (68), provide an alternative approach to screen suspected patients with OSA. It was observed that the convolutional neural network strategy exhibited the highest accuracy, sensitivity, and specificity among all existing algorithms. An alternative algorithm comprising optimized cardiovascular signals showed good representation of AHI and could be utilized to screen for OSA severity (69). Another study used six objective parameters including age, sex, body mass index, blood pressure, neck circumference, and the ESS and demonstrated the possibility for OSA screening and risk prediction (70).

OSA patients with cardiovascular conditions such as the heart failure and hypertension has been discussed before (71, 72), but their link to peripheral arterial disease (PAD) has been underestimated. The mechanism such as inflammation, oxidative stress, and endothelial dysfunction also contributed to the development of PAD in patients with OSA, and clinical evidences have been paid attention recently (73). Underdiagnosis of OSA in PAD were observed. A sleep apnea prevalence accounted for 78.0% in patients with lower extremity artery disease, and AHI increased with the severity of lower extremity artery disease (74). On the other hand, PAD prevalence of 98% in patients with confirmed OSA was observed (75). The association of OSA and PAD was further verified by a larger extent (76).

## Update on Treatment

Different approaches to treat OSA have been illustrated, and their influence on the AS and related diseases have gained considerable attention. The update treatment is discussed in detail in Table 2.

**Table 2:** Update on treatment.

**Method**	**Key points**		**References**
Continuous positive airway pressure (CPAP)	CPAP treatment was controversial regarding cardiovascular effects	Attenuate atherosclerosis	(79–82)
		Lack of clinical benefit in cardiovascular events	(83–85)
	Telemonitoring increased the CPAP adherence.		(87–91)
Mandibular advancement devices (MAD)	MAD treatment was controversial regarding cardiovascular effects.	Beneficial outcome in cardiovascular consequences	(80, 93)
		No effect on blood pressure and endothelial function	(94, 95)
	MAD may be an alternative compared to CPAP treatment		(80, 95, 96)
Exercise	Reduction of AHI, the increase of peak oxygen consumption		(98–102)
Hypoglossal nerve stimulation (HNS)	Beneficial for subjective and objective outcomes of sleep		(103–106)
Medications	Antioxidant		(108–110)
	Phenotype-based approach		(111–115)

### Continuous Positive Airway Pressure

Continuous positive airway pressure (CPAP) is widely used in the treatment of OSA to reduce excessive daytime sleepiness (77) and improve sleep quality (78). However, the cardiovascular effects of CPAP remain controversial. Some reports have supported that CPAP treatment attenuates AS (79). A further well-designed randomized controlled trial showed the modest ability of CPAP treatment to decrease blood pressure (80), indicating the potential to reduce cardiovascular morbidity and mortality. Patients with CAD also showed the attenuation with CPAP treatment. The risk of coronary heart disease in patients with OSA treated with CPAP was similar to those without OSA (81), and the risk of repeat revascularization under percutaneous coronary intervention was lowered when utilizing CPAP treatment (82). On the other hand, some evidence showed that CPAP therapy in preventing cardiovascular events lacked clinical benefit. A RCT showed that CPAP treatment did not significantly prevent hypertension or cardiovascular events (83). According to a large trial of sleep apnea cardiovascular endpoints in patients with OSA, additional CPAP treatment under usual care did not demonstrate the prevention of cardiovascular events (84). Most importantly, the average duration of CPAP should be paid attention to. In patients with CAD, CPAP had no significant effects on cardiovascular outcomes, but significant improvement was observed in patients with CPAP treatment for over 4 h (85). Therefore, improving CPAP compliance seems essential for treatment.

Many more studies have looked into CPAP adherence. Educational videos were utilized in patients with poor CPAP adherence and did not show superiority compared with usual care (86). In addition, remote telemonitoring for CPAP seems to be an effective alternative to improve adherence (87, 88). Text messages showed great improvement in medication compliance in chronic disease (89), and CPAP telemonitoring with automated feedback messages improved adherence in patients with OSA (88). The utilization of mobile applications associated with telemonitoring increase CPAP adherence with an average of 1 h (90). Notably, cardiovascular consequences in patients with OSA has been discussed under the CPAP treatment of remote patient telemonitoring. Given the large sample size of 306 patients, home self-measured blood pressure in patients with high cardiovascular risk did not show a significant difference between telemonitoring and usual care in CPAP treatment after 6 months (91). It was argued that telemonitoring focuses on CPAP without encouraging physical activity and thus, may not contribute to the reduction of blood pressure (91). However, the increase in CPAP adherence and the improvement of life quality was observed in favor of telemonitoring (91). Taken together, CPAP telemonitoring might allow better scrutiny of individual patient risks by connecting with devices measuring physical activity and blood pressure and further provide personalized care to patients with OSA and high cardiovascular risk.

### Mandibular Advancement Devices

Oral appliances have become an alternative way to treat OSA, especially mandibular advancement devices (MAD) (92). Some evidence has shown that MAD had a beneficial outcome in cardiovascular consequences. Compared with inactive control from the meta-analysis, MAD was associated with significant lower blood pressure in both systolic and diastolic blood pressure with a reduction of 2.1 and 1.9 mm Hg, respectively (80). Interestingly, it was reported that sex might affect the treatment with MAD. For women with MAD, the nighttime systolic and diastolic blood pressure was 10.8 and 6.6 mm Hg, respectively, lower than those in the sham group, while no significant differences were observed in men (93). However, the results comprised of 27 women and 58 men and might not present an unbiased conclusion. On the other hand, some reported that MAD had no effect on blood pressure and endothelial function although MAD improved the AHI, micro-arousal index and symptoms of fatigue, sleepiness and snoring (94). It was worth noting that patients included in this study had severe OSA without overt cardiovascular disease, which could not provide an objective result. Another study also failed to show significant beneficial effect on the endothelial function and sleep-time blood pressure under MAD treatment in patients with 20–40/h of AHI (95). That is, the cardiovascular outcome of MAD treatment remained unclear and needs further investigation.

In contrast to CPAP, the clinical effectiveness of MAD treatment was not good enough in terms of AHI reduction, but the cost effectiveness was better considering quality-adjusted life-years based on the questionnaire in moderate OSA (96). As for cardiovascular outcome, systolic and diastolic blood pressure showed no significant differences in both treatments according to the meta-analysis (80). Additionally, endothelial function, and sleep-time blood pressure showed similar performances (95). In conclusion, MAD may be a good alternative approach for patients refusing CPAP therapy or for those who prefer MAD due to the less-invasive nature of the device.

### Exercise

Reduced exercise capacity was observed in patients with OSA, and comorbidities such as, daytime hypoxemia and severe mean nocturnal desaturation made the exercise capacity lower (97). Further, exercise training contributed to either decrease of AHI (98) or reduction of body weight (99) in patients with OSA. Recent studies emphasized the importance of exercise treatment, and evidence showed exercise would bring a great improvement in patients with OSA and cardiovascular diseases. In all, 35% (100) and 33% (101) reduction of AHI was observed in OSA patients with heart failure and CAD, respectively, after exercise treatment. In addition, peak oxygen consumption, muscle strength, and endurance greatly improved with exercise treatment instead of CPAP (100) in patients with OSA and heart failure, which have important clinical implications. The performance of increasing peak oxygen consumption was also observed in patients with OSA and CAD (102). Therefore, exercise is an important therapy for patients with OSA, especially with cardiovascular comorbidity.

### Hypoglossal Nerve Stimulation

Hypoglossal nerve stimulation devices are used to dilate/reinforce the airway by neuromodulation. A case report showed that in a patient with unsuccessful surgery, HNS treatment drastically improved their condition and outcome (103). Then, long-time HNS treatment with 48 months were investigated, and stable improvement was observed among 91 patients with moderate-to-severe OSA (104). Alternation of the nocturnal sleep architecture and improvement of the objective level of alertness were also reported after HNS therapy (105). However, the side effect of pain, tongue abrasion, and device malfunction should be noted for further use (106).

### Drug Therapy

Some medications have recently been investigated for OSA. As oxidative stress is an aspect of OSA, antioxidants was designed for treatment (107–109). Losartan, an antioxidant, and anti-inflammatory drug (108), showed a significant effect in the treatment of cardiovascular complications in OSA (109). Another therapeutic candidate is melatonin, a regulating hormone with antioxidant properties, which showed its cardioprotective effects on myocardial injuries (110). Taken together, antioxidants might be considered as a prospective drug in patients with OSA and cardiovascular diseases.

Some drug therapies have focused on the phenotype in OSA, such as, high loop gain, pharyngeal hypotonia, and low arousal threshold (111). Oxygen could be utilized for downregulation of the loop gain. Thirty-six patients with an average AHI of 57.9 events/h accomplished two nights of polysomnography in favor of supplemental oxygen (40%) and sham (air). Nine of the patients exhibited a reduction of 70% in AHI and 7 mm Hg overnight change in blood pressure (112). The decrease of pharyngeal dilator muscle activity during sleep is one of the key factors resulting in upper airway collapse. It was reported that desipramine, a noradrenergic agonist, improved pharyngeal collapsibility, and might be a new pharmacologic therapy for patients with OSA (113, 114). In addition, it was observed that the respiratory arousal threshold could be increased by drugs such as, trazodone, but it was insufficient to improve the compromised upper airway anatomy (115). However, drug-induced prolonged arousal trait might contribute to the undesirable high loop gain trait, which could be hazardous in patients with cardiovascular diseases. The precision-medicine approach targeting the phenotypic traits of OSA showed a fair degree of uncertainty and thus, more research should be undertaken to comprehensively investigate its safety and efficacy.

## Conclusion and Prospect

Many studies have provided descriptions of patients with OSA and AS-associated cardiovascular diseases. Intermittent hypoxia, an important characteristic of OSA, facilitates the occurrence, and development of AS. The mechanism discussed in this review have not develop into diagnosis and therapy. Although, some proteins and molecules showed its distinct characteristics in the progress, they almost remained in a cell or mouse model without the support of human experiments. In addition, the relationship between IH and AS is extremely complex and the results from *in vitro* and *in vivo* studies are sometimes inconsistent. Thus, signaling processes need further exploration, and the comprehensive theory is also required for better diagnosis and treatment.

Updated diagnosis and treatment methods are summarized in Tables 1, 2, except for the gold standard polysomnography and CPAP. Since the relationship of PAD and OSA was underestimated in the diagnosis and treatment, much more attention should be paid in the further research. The development of portable device for diagnosis gave the possibility to cover more and more patients with OSA especially for those without hospitalization, and it is much more convenient and cost-efficient. Additionally, treatments are well-discussed, and their effect on AS and related cardiovascular diseases are not clear. For example, the clinical effect of CPAP and MAD on cardiovascular diseases is controversial. Therefore, large-scale and long-term clinical studies with a robust scientific design are crucial for the comparison of different methods that contribute to diagnosis and treatment of acute apnea syndrome with AS and related diseases.

## Author Contributions

JC drafted the manuscript. YZ and SL supervised and revised it. All authors contributed to the article and approved the submitted version.

## Conflict of Interest

The authors declare that the research was conducted in the absence of any commercial or financial relationships that could be construed as a potential conflict of interest.
